# Transanal Mesenteric Resection in Hirschsprung's Disease Using ICG under Concept of NOTES Technique

**DOI:** 10.1055/s-0042-1751051

**Published:** 2022-08-16

**Authors:** Mitsuru Muto, Shun Onishi, Masakazu Murakami, Keisuke Yano, Toshio Harumatsu, Satoshi Ieiri

**Affiliations:** 1 Department of Pediatric Surgery, Kagoshima University, Kagoshima, Japan

**Keywords:** Hirschsprung's disease, laparoscopic-assisted transanal endorectal pull-through, NOTES, mesenteric processing, indocyanine green

## Abstract

Laparoscopic surgery has been applied for Hirschsprung's disease (HD). We herein report our approach to mesenteric processing for laparoscopic-assisted transanal endorectal pull-through (L-TERPT). Following mucosectomy and entering the abdominal cavity, a vessel sealing system is transanally inserted into the abdominal cavity for mesenteric processing based on concept of Natural Orifice Translumenal Endoscopic Surgery. Since the transanal axis is parallel to the dissected mesentery, it makes easier to operate in comparison to when the procedure is performed through the abdominal working port and can reduce the additional abdominal trocar wound. We also use indocyanine green (ICG) fluorescence navigation. Fluorescing the vessels with ICG allows intraoperative visualization of the blood flow in the retrieved intestine. With these innovative combined techniques, L-TERPT for HD can be safely performed, even in infants with small intraabdominal cavities.

## Introduction

Since the 1990s, Smith BM, 1 Georgeson KE, 2 Hoffmann K, 3 Rothenberg and Chang, 4 and others have introduced laparoscopic procedures in the treatment of Hirschsprung's disease (HD). We perform laparoscopic-assisted endorectal pull-through (L-TERPT) for patients with a body weight of around 4 kg. In this technical manuscript, we report on our innovative approach to mesenteric processing in HD based on the concept of Natural Orifice Translumenal Endoscopic Surgery (NOTES). 5

## Case Report



**Video 1** Additional supporting information, our innovative combined technique video on laparoscopic-assisted transanal endorectal pull-through, may be found online in the Supporting Information section at the end of this article.


A male infant presented with abdominal distension on the third day of life. An enema showed a caliber-change in his sigmoid colon, and a rectal mucosal suction biopsy revealed hyperplasia of exogenous nerve fibers. A preoperative diagnosis of rectosigmoid type HD was made. At 4 months of age, he weighed 4,870 g and underwent L-TERPT.

**Fig. 1:**
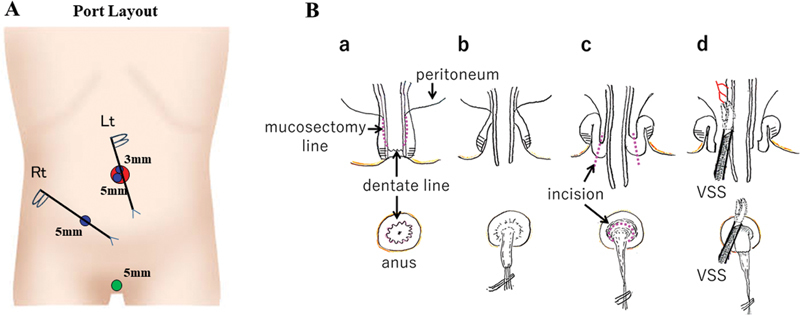
( **A** ) Port layout. Port layout is shown. ( **B** ) Schema for the mucosectomy and mesenteric dissection. The mucosectomy was stared at 1 cm from the dentate line ( **a** ). Some sutures were applied to the removing mucosa, and mucosectomy was performed to the oral side with traction ( **b** ). The dissection was advanced to the level of the peritoneal reflection, and the circumferential incision was made at the folded muscle to reach the peritoneal cavity ( **c** ). A vessel sealing system (VSS) was inserted into the abdominal cavity through the incision, and the mesentery of the pull-through intestine was coagulated and divided ( **d** ) while checking the blood flow with indocyanine green under observation by a laparoscope.

**Fig. 2:**
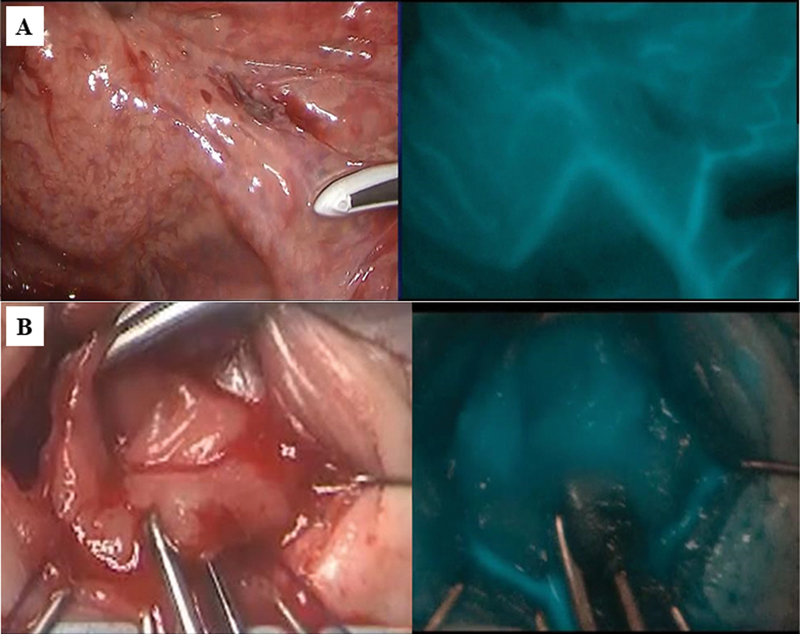
( **A** ) Mesenteric blood flow assessment. The mesentery blood flow in the pulled-through sigmoid colon was confirmed with indocyanine green (ICG) fluorescence. ( **B** ) Evaluation of blood flow in the anastomotic sigmoid stump. Sufficient blood supply in the pulled through sigmoid colon was confirmed with ICG fluorescence.

The patient was placed in the lithotomy position. A longitudinal incision was made in the umbilicus and multichannel access device (LAPPROTECTOR-minimini and EZ-access, Hakko, Tokyo, Japan) was attached, then a camera port (5-mm) and operator's left-hand port (3-mm) were placed. After pneumoperitoneum was established under 7 mm Hg with 3 L/min CO _2_ , a 5-mm 30-degree laparoscope was inserted, and the operator's right-hand port (5-mm) was placed in the lower-right abdomen ( Fig. 1A ). The definitive diagnosis of HD was made by intraoperative laparoscopic full-thickness biopsy and the location of sigmoid colon with normal ganglion cells was identified. Mucosectomy was started at 1 cm cranial side from the dentate line. A circumferential incision of the peritoneal reflection was made. Then, entering abdominal cavity through the anus, a 5-mm vessel sealing system (VSS; Ligasure-Maryland, Medtronic, MN, United States) was inserted transanally into the abdominal cavity under observation by a laparoscope, to dissect the mesentery of the narrow segment of the colon ( Fig. 1B ). Indocyanine green (ICG, 25 mg [Diagnogreen, Daiichi Sankyo Company, Limited, Tokyo, Japan]) was dissolved in 10 mL of distilled water. The mesentery blood flow in the pulled-through colon was confirmed under a fluorescence scope, with the intravenous administration of 1 mL (0.5 mg/kg) of ICG solution ( Fig. 2A ). Soave procedure is applied as our standard approach. After partial resection of the dorsal muscle cuff, the normal colon was pulled through to the perineum. Sufficient blood supply in the pulled-through colon was also confirmed from the perineal side with ICG fluorescence again ( Fig. 2B ), and anal anastomosis was made with 5–0 monofilament sutures ( Video 1 ). Postoperative defecation function was well maintained with support of enemas every 2 days.

## Discussion

Preserving the marginal artery while processing the mesentery is the most important with L-TERPT to maintain sufficient blood flow at the pulled-through bowel stump. ICG fluorescence navigation, which has been reported to be useful in the field of gastrointestinal surgery in recent years, 6 7 provides intraoperative visualization and recognition of the retention of intestinal blood flow during anastomosis. When processing of the mesentery of the pulled-through bowel is required, we insert a VSS transanally. This concept is similar to that of NOTES, 5 in which the abdominal cavity is reached and surgery is performed through the gastrointestinal tract. In comparison to the axis on the operator's right hand, the transanal axis is parallel to the mesentery; hence, we believe it facilitates easier vessel processing. The advantages of this method are good visibility and ease of operation. We have no cases of intra-abdominal infection caused by contamination due to the transanal manipulation. With these innovative combined techniques, L-TERPT can be safely performed even in small infants.

## Conclusion

The concept of NOTES can reduce the operative scar of the body surface. Transanal device insertion and mesenteric processing in TERPT procedure for HD after entering abdominal cavity is reasonable technique. ICG allows intraoperative visualization of the blood flow in the retrieved intestine. With these innovative combined techniques, L-TERPT for HD can be safely performed, even in infants with small intra-abdominal cavities.

## Notes

New Insight and the Importance for the Pediatric Surgeon The concept of NOTES can reduce the operative scar of the body surface. Transanal device insertion and mesenteric processing in TERPT procedure for HD after entering abdominal cavity is reasonable technique.
